# Characterization of the complete chloroplast genome of *Ottelia alismoides* (Hydrocharitaceae), a vulnerable submerged macrophyte in China

**DOI:** 10.1080/23802359.2019.1704197

**Published:** 2020-01-21

**Authors:** Jian-Ling Guo, Yan-Hong Yu, Yong-Hong Zhang

**Affiliations:** a School of Life Sciences, Yunnan Normal University, Kunming, China;; b Key Laboratory for Pollution Process and Watershed Management of Plateau Lakes in Yunnan Province, Yunnan Institute of Environmental Science, Kunming, China

**Keywords:** *Ottelia alismoides*, chloroplast genome, submerged macrophyte endangered species

## Abstract

*Ottelia alismoides* (Linn.) Pers. (Hydrocharitaceae) is an endangered submerged macrophyte in China. In his study, we assembled complete chloroplast (cp) genome of *O. alismoides* based on the Illumina reads. The cp genome of *O. alismoides* was 157,885 bp in length, including a large single copy (LSC) region of 87,707 bp and small single copy (SSC) region of 20,234 bp, separated by two inverted repeat (IR) regions of 24,972 bp each. The cp genome encoded 115 genes including 70 protein-coding genes, 37 tRNA genes, and 8 rRNA genes. The GC content of cp genome of *O. alismoides* is 36.6%. A maximum likelihood phylogenetic analysis revealed that *O. alismoides* is closely related to *O. acuminate* var. *songmingensis*.

*Ottelia alismoides* (Linn.) Pers., a submerged macrophyte of Hydrocharitaceae, is widely distributed in the tropical and warmer regions of Asia, Australasia and a part of Africa (Cook and Urmi-König 1984). In China, *O. alismoides* occurs in lake shorelines, marsh ponds, irrigation ditches and stream margins in water ranging from 0.05 to 1 m deepth (He 1991). *O. alismoides* lacks specialized organs for vegetative reproduction. Population recruitment and expansion depends solely on sexual reproduction (Jiang and Kadono 2001). Its populations have decreased rapidly during the past two decays, and it has been categorized as vulnerable with a status ‘A2ac’ in the China Species Red List (Yu et al. 1998; Qin et al. 2017). Until now, fundamental genetic information about this endangered aquatic macrophyte remains less. Here, the complete chloroplast genome of *O. alismoides* was characterized to provide genomic resource for further conservation genetics and phylogenetic analysis.

The fresh leaves of *O. alismoides* were collected from Huangmei town, Jurong City of Jiangsu Province (32°02′59″N, 119°08′24″E), and the voucher specimen(Guo AL01) was deposited at Herbarium of Yunnan Normal University. A sequence library for *O. alismoides* was generated using the Illumina HiSeq 2500-PE150 platform (Illumina, San Diego, CA). High-quality clean reads were obtained using NGS QC Toolkit_v2.3.3 with default parameters (Patel and Jain 2012). The plastome was de novo assembled using NOVOPlasty (Dierckxsens et al. 2017). The cp genome was annotated with the online annotation tool GeSeq (Tillich et al. 2017).

The total chloroplast genome size of *O. alismoides* is 157,885 bp (GenBank accession no. MK922145), containing a large single copy (LSC) region of 87,707 bp and a small single copy (SSC) region of 20,234 bp, separated by two inverted repeat (IR) regions of 24,972 bp. It contained 115 genes, including 70 protein-coding, 8 rRNA genes, and 37 tRNA genes. The base compositions of the chloroplast genome were uneven (31.3% A, 18.7% C, 17.9% G, 32.1% T), with an overall GC content of 36.6%. Total 76 simple sequence repeats (SSRs) were discovered using the online software IMEx (Mudunuri and Nagarajaram 2007). Among them, the numbers of mono-, di-, tri-, tetra-, penta and hexa- nucleotides SSRs are 32, 28, 2, 12, 2 and 0, respectively.

To clarify the phylogenetic position of *O. alismoides*, the published chloroplast genomes, including *O. acuminate var. songmingensis* (MK604184, Guo et al. 2019) and *Elodea canadensis* (NC_018541, Huotari and Korpelainen 2012) from Hydrocharitaceae and 15 species from Alismatales, with 3 *Acorus* species from Acorales as outgroups, were aligned by MAFFT 7.308 (Katoh and Standley 2013). The maximum likelihood (ML) tree was constructed with RAxML version 8 (Stamatakis 2014) and the clade support was estimated using 1000 bootstrap replicates. The phylogenetic tree showed that three species of Hydrocharitaceae formed one monophyletic clade with 100% bootstrap value (Figure 1) and *O. alismoides* was closely related with its congeneric species, *O. acuminate* var. *songmingensis*. The complete chloroplast genome of *O. alismoides* will provide useful resource for further study on phylogeny, conservation genetics and molecular breeding.

**Figure 1.**
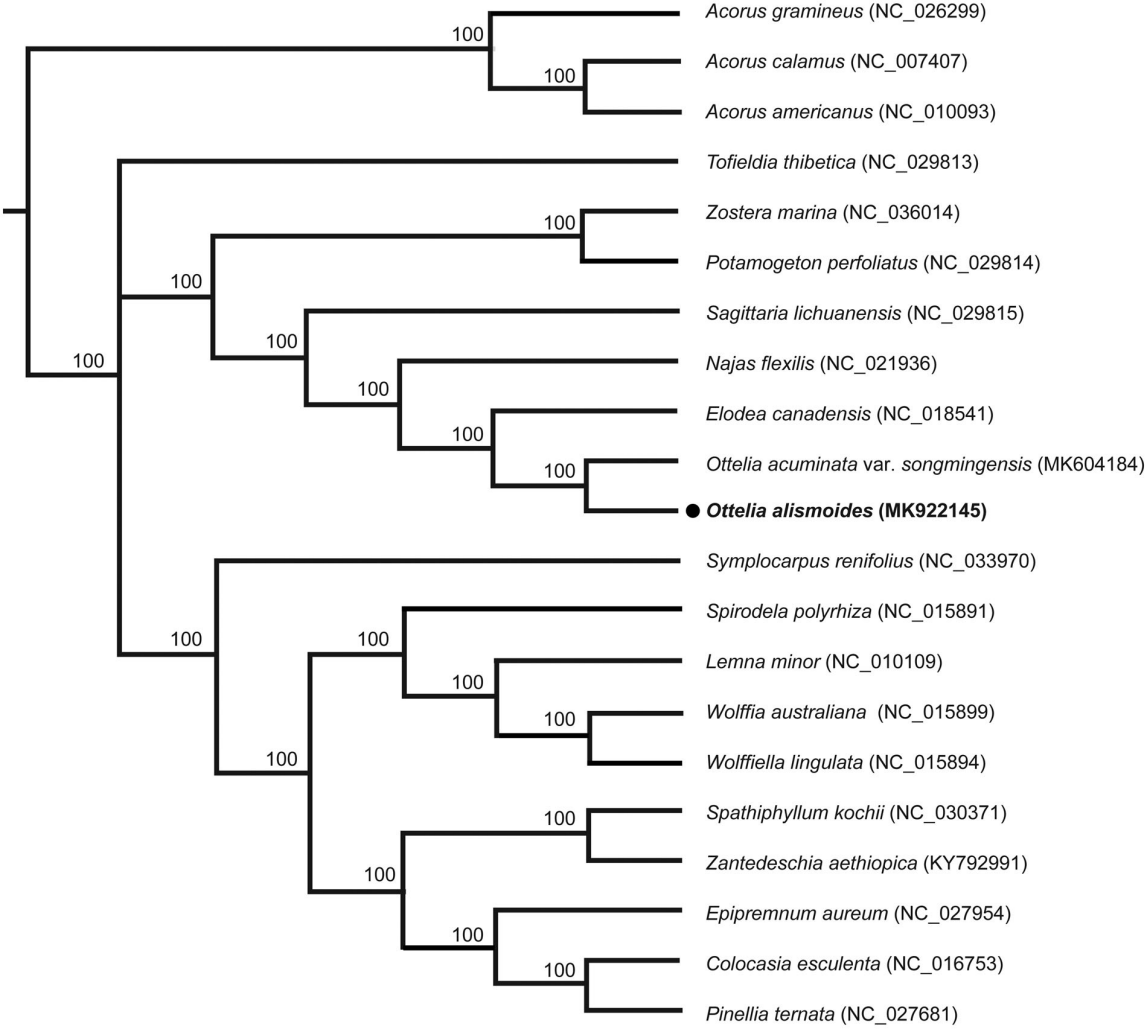
Phylogenetic tree of 21 samples using maximum likelihood based on complete chloroplast genome. Bootstrap support values >50% are indicated next to the branches.
